# Clinical Characteristics of EGPA Patients in Comparison to GPA Subgroup with Increased Blood Eosinophilia from POLVAS Registry

**DOI:** 10.1155/2024/4283928

**Published:** 2024-04-25

**Authors:** Anna Drynda, Agnieszka Padjas, Krzysztof Wójcik, Radosław Dziedzic, Grzegorz Biedroń, Katarzyna Wawrzycka-Adamczyk, Anna Włudarczyk, Joanna Wilańska, Jacek Musiał, Zbigniew Zdrojewski, Zenobia Czuszyńska, Anna Masiak, Maria Majdan, Radosław Jeleniewicz, Hanna Augustyniak-Bartosik, Katarzyna Jakuszko, Magdalena Krajewska, Alicja Dębska-Ślizień, Hanna Storoniak, Barbara Bułło-Piontecka, Witold Tłustochowicz, Joanna Kur-Zalewska, Małgorzata Wisłowska, Piotr Głuszko, Marta Madej, Ewa Jassem, Iwona Damps-Konstańska, Eugeniusz Kucharz, Marek Brzosko, Marcin Milchert, Anna Hawrot-Kawecka, Joanna Miłkowska-Dymanowska, Paweł Górski, Anna Lewandowska-Polak, Joanna Makowska, Joanna Zalewska, Lech Zaręba, Stanisława Bazan-Socha

**Affiliations:** ^1^Students' Scientific Group of Immune Diseases and Hypercoagulation, Jagiellonian University Medical College, Cracow, Poland; ^2^2nd Department of Internal Medicine, Jagiellonian University Medical College, Cracow, Poland; ^3^Doctoral School of Medical and Health Sciences, Jagiellonian University Medical College, Cracow, Poland; ^4^Department of Internal Medicine, Connective Tissue Diseases and Geriatrics, Medical University of Gdansk, Gdansk, Poland; ^5^Department of Rheumatology and Connective Tissue Diseases, Medical University of Lublin, Lublin, Poland; ^6^Department of Nephrology and Transplantation Medicine, Wroclaw Medical University, Wroclaw, Poland; ^7^Department of Nephrology, Transplantology and Internal Diseases, Medical University of Gdansk, Gdansk, Poland; ^8^Department of Internal Medicine and Rheumatology, Military Medical Institute, Warsaw, Poland; ^9^Department of Rheumatology, National Institute of Geriatrics, Rheumatology and Rehabilitation, Warsaw, Poland; ^10^Department of Rheumatology and Internal Medicine, Wroclaw Medical University, Wroclaw, Poland; ^11^Department of Allergology, Medical University of Gdansk, Gdansk, Poland; ^12^Department of Internal Medicine, Rheumatology and Clinical Immunology, Medical University of Silesia, Katowice, Poland; ^13^Department of Rheumatology, Internal Medicine, Diabetology, Geriatrics and Clinical Immunology with the Gastroenterology Unit, Pomeranian Medical University in Szczecin, Szczecin, Poland; ^14^Department of Internal Medicine and Metabolic Diseases, Medical University of Silesia, Katowice, Poland; ^15^Department of Pneumology, Chair of Internal Medicine, Medical University of Lodz, Lodz, Poland; ^16^Department of Rheumatology, Medical University of Lodz, Lodz, Poland; ^17^Department of Rheumatology and Connective Tissue Diseases, Ludwik Rydygier Collegium Medicum in Bydgoszcz of the Nicolaus Copernicus University in Torun, Bydgoszcz, Poland; ^18^Institute of Computer Science, College of Natural Sciences, University of Rzeszow, Rzeszow, Poland

## Abstract

**Objective:**

To characterize the eosinophilic granulomatosis with polyangiitis (EGPA) population from the POLVAS registry depending on ANCA status and diagnosis onset, including their comparison with the granulomatosis with polyangiitis (GPA) subset with elevated blood eosinophilia (min. 400/*μ*l) (GPA HE) to develop a differentiating strategy.

**Methods:**

A retrospective analysis of the POLVAS registry.

**Results:**

The EGPA group comprised 111 patients. The ANCA-positive subset (*n* = 45 [40.54%]) did not differ from the ANCA-negative one in clinics. Nevertheless, cardiovascular manifestations were more common in ANCA-negative patients than in those with anti-myeloperoxidase (MPO) antibodies (46.97% vs. 26.92%, *p* = 0.045). Patients diagnosed before 2012 (*n* = 70 [63.06%]) were younger (median 41 vs. 49 years, *p*  < 0.01), had higher blood eosinophilia at diagnosis (median 4,946 vs. 3,200/*μ*l, *p*  < 0.01), and more often ear/nose/throat (ENT) and cardiovascular involvement. GPA HE comprised 42 (13.00%) out of 323 GPA cases with reported blood eosinophil count. Both GPA subsets had a lower prevalence of respiratory, cardiovascular, and neurologic manifestations but more often renal and ocular involvement than EGPA. EGPA also had cutaneous and gastrointestinal signs more often than GPA with normal blood eosinophilia (GPA NE) but not GPA HE. The model differentiating EGPA from GPA HE, using ANCA status and clinical manifestations, had an AUC of 0.92, sensitivity of 96%, and specificity of 95%.

**Conclusion:**

Cardiovascular symptoms were more prevalent in the ANCA-negative subset than in the MPO-ANCA-positive one. Since EGPA and GPE HE share similarities in clinics, diagnostic misleading may result in an inappropriate therapeutic approach. Further studies are needed to optimize their differentiation and tailored therapy, including biologics.

## 1. Introduction

Eosinophilic granulomatosis with polyangiitis (EGPA), formerly Churg–Strauss syndrome, is a rare form of vasculitis affecting small to medium blood vessels. It is characterized by eosinophil rich, necrotizing granulomatosis inflammation, and a substantial increase in blood and tissue eosinophilia. Extravascular inflammation, both granulomatous and nongranulomatous, can involve various tissues, leading to diverse clinical manifestations [1].

Despite the fact that EGPA is classified as a subtype of anti-neutrophil cytoplasmic antibody (ANCA)-associated vasculitis (AAV), the ANCA-positive subset accounts for only 30%–40% of cases [2–4]. A perinuclear pattern of ANCA in IIF, associated predominantly with anti-myeloperoxidase (MPO-ANCA) antibodies, is observed in the majority of ANCA-positive EGPA cases [5].

In EGPA, two main clinical phenotypes are based on ANCA status [6]. However, some clinical features, such as adult-onset asthma, recurrent or chronic rhinosinusitis, nasal polyps, and, occasionally, life-threatening alveolar hemorrhage, may prevail in both subsets. On the other hand, certain manifestations are attributed to ANCA-negative EGPA, indicating the eosinophilic tissue infiltration, or the ANCA-positive EGPA, where the vasculitis plays a pivotal role. The former is characterized by cardiac involvement, encompassing cardiomyopathy with left ventricular dysfunction, valvular insufficiency, conduction defects, and pericardial effusion, parallel with gastrointestinal signs. Skin lesions associated with ANCA-positive EGPA manifest as either hemorrhagic, predominantly presenting as palpable purpura, or as dermal or subcutaneous nodules and papules. Other manifestations closely related to vasculitis comprise peripheral neuropathy and glomerulonephritis [6–9].

The American College of Rheumatology (ACR) in 1990 proposed classification criteria for Churg–Strauss syndrome comprising six clinical features, including eosinophilia >10%, mono- or poly-neuropathy, nonfixed pulmonary infiltrates, paranasal sinus abnormality, and extravascular eosinophils [10]. At least four criteria must be met to classify the case as EGPA. However, the current nomenclature published in 2012 [1] and revised in 2017 [7] refers to the patients with asthma and hypereosinophilia (i.e., circulating eosinophilia ≥1,500/*μ*l and/or ≥10% of white blood cells) who fulfill at least one of the definite features or surrogates of vasculitis, or ANCA presence with any systemic organ involvement. While the currently utilized approach is more sensitive [11], it is essential to note that vasculitis-affiliated centers now treat patients diagnosed before and after 2012. Therefore, comparing patients diagnosed in different timeframes may provide valuable insights.

Interestingly, hypereosinophilia was also documented in granulomatosis with polyangiitis (GPA) [12]. Hence, for individuals diagnosed with small- or medium-vessel polyangiitis who also exhibit an elevated blood eosinophil count, it may be beneficial to consider the currently proposed classification criteria for vasculitis by the ACR and the European Alliance of Associations for Rheumatology (EULAR). These criteria primarily rely on factors such as respiratory and kidney involvement, blood eosinophilia, and anti-proteinase 3 (PR3) antibody status to distinguish EGPA from other polyangiitis [13]. However, they were not developed for diagnostic purposes or validated in prospective clinical trials.

So far, the treatment of choice for remission induction in new onset or relapsing EGPA consists of systemic glucocorticosteroids (GCs) or the immunosuppressant–glucocorticoid combination, depending on the leading clinical manifestation [14]. Since those therapeutic approaches are associated with severe adverse effects and often do not meet expectations in terms of outcomes, biological treatments have been introduced, mainly rituximab and mepolizumab [15]. Other treatments, such as benralizumab, are currently under investigation in EGPA [15]. The emergence of new therapies can create a dilemma of choice, which could be addressed by stratifying patients to those who would benefit most [16]. However, further clinical and laboratory investigations, including pathogenetic disease characteristics, are needed.

Therefore, this study aimed to characterize the EGPA population from the POLVAS registry. This encompassed a comparative analysis of subsets with varying ANCA status to ascertain whether they exhibit distinct phenotypes. Furthermore, the study sought to investigate how patient characteristics and treatment approaches have evolved over time by considering the onset of diagnosis. Additionally, we conducted a comparative analysis between EGPA and GPA with elevated blood eosinophilia to look for potential similarities that may inform future diagnostic processes and treatment strategies.

## 2. Materials and Methods

### 2.1. Patients

The low prevalence of vasculitides makes data collection difficult; hence, in 2017, the Scientific Consortium of the Polish Vasculitis Registry (POLVAS) was created, including 10 academic centers for treating systemic vasculitides. It facilitates retrospective data collection on clinical features, treatments, and outcomes of Polish white Caucasian patients affected by those diseases [17, 18].

The registry encompasses 111 EGPA patients with clinical data sufficient to confirm the diagnosis, all of whom were diagnosed from 1998 to 2020 and stayed under the care of physicians from POLVAS-affiliated centers.

Although there are no diagnostic criteria for EGPA in the strict sense, the diagnosis in all patients was confirmed using the ACR 1990 classification criteria [10] and the nomenclature proposed by the 2012 Revised International Chapel Hill Consensus [1]. Since the latter was published in 2012 [1], patients in our analysis were divided into two subgroups: (1) diagnosed in 2012 and (2) diagnosed after 2012.

Due to the distinct characteristics of ANCA-positive and ANCA-negative subsets, patients were also divided according to ANCA status. We performed a separate comparison of MPO-ANCA-positive and ANCA-negative subtypes as well.

Additionally, 42 patients with GPA included in the POLVAS registry were characterized by blood eosinophilia at diagnosis ≥400/*μ*l, classified as increased blood eosinophilia. Among them, 23 patients filled the criterion of hypereosinophilia defined as peripheral blood eosinophil count ≥1,500/*μ*l or >10% of total white blood cells. The remaining 281 GPA patients with known blood eosinophilia at diagnosis were also included in the analysis as a separate subset.

Regarding microscopic polyangiitis (MPA), only 10 patients with increased blood eosinophilia were reported in the POLVAS registry.

### 2.2. The POLVAS Registry Construction

To enable reporting symptoms, each of them was classified into one of the broader categories. Details on symptoms category manifestations are provided in Supplementary Data S1.

All presented symptoms were treated as vasculitis related if no other explanations were found after a detailed and thorough diagnosis.

### 2.3. Statistical Analysis

The normal distribution of continuous variables was verified by the Shapiro–Wilk test. All data were non-normally distributed; thus, they were presented as median and 0.25–0.75 interquartile range and compared by the Mann–Whitney *U*-test. Categorical variables were given as numbers and percentages and compared using the *χ*^2^ test, with Yates correction, adjusted for age and gender if needed. Statistical significance was defined as a two-tailed *p*-value of <0.05. The algorithm developed to distinguish the patients with EGPA from the patients with GPA and elevated eosinophil blood count was based on the classification and regression tree (CART) method. Receiver operating characteristic (ROC) analysis was employed to assess the performance of the obtained model. Calculations were made using StatSoft Statistica 13.3 (TIBCO Software, Palo Alto, USA) and R 4.3.1 (R Foundation for Statistical Computing, Vienna, Austria) software.

## 3. Results

### 3.1. Anti-Neutrophil Cytoplasmic Antibodies (ANCA)-Positive Eosinophilic Granulomatosis with Polyangiitis (EGPA) Patients Were Older at Diagnosis but Had Similar Clinical Manifestations than ANCA-Negative Subset

A comparison of ANCA-positive and ANCA-negative subsets is presented in Table 1.

There were *n* = 45 ANCA-positive cases (40, 54%) who were older at diagnosis but did not differ in clinical manifestations, exacerbation rate, treatment mode, highest blood eosinophilia, and CRP levels (Table 1). As expected, the most prevalent in both subsets were respiratory symptoms documented in over 95% of cases (95.56% vs. 96.97%), followed by constitutional (88.89% vs. 89.39%) and ear/nose/throat (ENT) signs (84.44% vs. 81.82%).

Among treatment modalities, in the induction remission phase, in both subsets, the most common were oral GCs (93.33% vs. 92.42%), intravenous methylprednisolone pulses (60.00% vs. 71.21%), and cyclophosphamide (40.0% vs. 46.97%), while in maintenance treatment, oral GCs (71.11% vs. 56.06%), azathioprine (35.56% vs. 27.27%), and methotrexate (31.11% vs. 21.21%).

### 3.2. Cardiovascular Symptoms Were More Prevalent in Anti-Neutrophil Cytoplasmic Antibodies (ANCA)-Negative Eosinophilic Granulomatosis with Polyangiitis (EGPA) Patients than in Those with Anti-Myeloperoxidase Antibodies (MPO-ANCA) Presence

**Figure 1:**
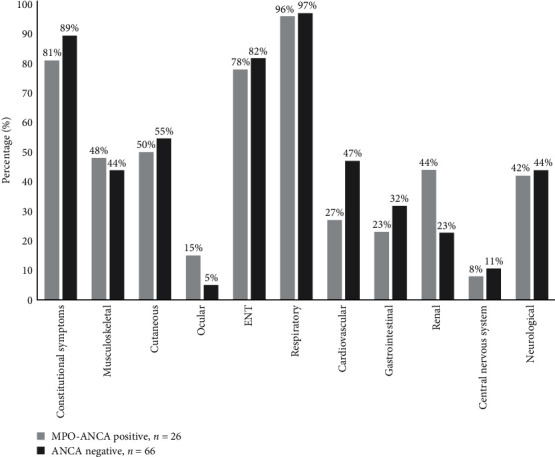
Clinical manifestations in MPO-ANCA-positive and ANCA-negative EGPA subsets. Categorical variables are presented as percentages. Abbreviations: ANCA, anti-neutrophil cytoplasmic antibodies; MPO-ANCA, anti-myeloperoxidase antibodies; and ENT, ear/nose/throat.

An additional comparison of MPO-ANCA-positive and ANCA-negative subsets is depicted in Figure 1.

Interestingly, as shown in Figure 1, cardiovascular manifestation was 20% more prevalent in ANCA-negative patients than in MPO-ANCA-positive ones (46.97% vs. 26.92%, *p* = 0.045). On the other hand, renal involvement was 20% more common in the MPO-ANCA-positive subtype than in the ANCA-negative (44.00% vs. 22.72%, Figure 1), although the difference did not reach significance (*p* = 0.07). Likewise, gastrointestinal symptoms were slightly more prevalent but insignificant (*p* = 0.3) in ANCA-negative patients (Figure 1).

### 3.3. Eosinophilic Granulomatosis with Polyangiitis (EGPA) Patients Diagnosed According to the 1990 American College of Rheumatology Classification Criteria Were Younger at Diagnosis, Had More Prevalent Ear/Nose/Throat and Cardiovascular Manifestations, Higher Blood Eosinophilia, and Increased Exacerbation Rate in Follow-Up

Detailed demographic and clinical characteristics of patients stratified according to the year of diagnosis; thus, different criteria are shown in Table 2.

Patients diagnosed according to 1990 ACR criteria were a median of 8 years younger at diagnosis than those diagnosed after 2012 (41.3 vs. 49.17 years, *p*  < 0.01) (Table 2). Furthermore, they were characterized by over 20% more prevalent ENT (91.43% vs. 68.29%, *p*  < 0.01) and cardiovascular (50.00% vs. 29.27%, *p* = 0.04) symptoms and had higher exacerbation rates and 1.5-fold higher maximal peripheral blood eosinophilia (4,946 vs. 3,200/*μ*l, *p*  < 0.01).

Interestingly, GCs were the mainstay of treatment until 2012. On the contrary, later, immunosuppressant use increased in favor of GCs reduction.

According to the biologics and other immunosuppressive approaches, in our cohort, only one patient received benrolizumab and two intravenous immunoglobulins.

### 3.4. The Granulomatosis with Polyangiitis (GPA) Subgroup with Increased Blood Eosinophilia (*n* = 42) Had Similar Age at Diagnosis and Prevalence of Cutaneous and Gastrointestinal Manifestations but More Common Renal and Ocular Involvement than Eosinophilic Granulomatosis with Polyangiitis (EGPA) Patients

Only 23 (7.12%) out of 323 GPA patients with a known blood eosinophil count in the POLVAS registry filled the criterion for hypereosinophilia, and an additional 19 cases (5.88%) had increased blood eosinophil count in the range of 400–1,500 cell/*µ*l. All 42 were classified as GPA with increased blood eosinophilia (GPA HE). Detailed characteristics of EGPA patients in comparison to GPA HE and GPA with blood eosinophils count <400/*µ*l (GPA NE) are presented in Table 3.

EGPA and GPA HE patients did not differ in age of diagnosis, but both those groups were younger than GPA NE (*p*  < 0.01 and *p* = 0.03, respectively, Table 3). The percentage of females was also different, being over 15% and 31% lower in GPA NE and GPA HE than EGPA (*p* = 0.03 and *p*  < 0.01, respectively).

GPA HE and GPA NE did not differ in clinics. In turn, the EGPA group had more common respiratory, cardiovascular, and neurological manifestations but fewer renal and ocular signs than both GPA subsets. Furthermore, EGPA patients had more often cutaneous and gastrointestinal symptoms than GPA NE, but not GPA HE (Table 3)

The flare-up rate was higher in EGPA than in both GPA subsets; however, severe exacerbations, i.e., requiring hospitalization, were less prevalent in those patients (Table 3).

As expected, both GPA subgroups achieved higher doses of steroids and cyclophosphamide than EGPA in the remission induction phase and the maintenance therapy. Methotrexate, on the other hand, was more often prescribed in EGPA patients. Only in GPA rituximab and blood apheresis were applied.

EGPA and GPA groups also differed in terms of laboratory parameters.

Both GPA subsets were more commonly PR3-ANCA positive, although that type of antibody was also documented in about 10% of EGPA patients. On the other hand, MPO-ANCA was recorded in 23.42% of EGPA and 5.88% of GPA patients (*p*  < 0.01).

The EGPA group was characterized by over sevenfold (4,992 vs. 700/*μ*l, *p*  < 0.01) and 50-fold (4,992 vs. 100/*μ*l, *p*  < 0.01) higher median blood eosinophilia at diagnosis than GPA HE and GPA NE, respectively. On the other hand, maximal C-reactive protein was higher in GPA patients (Table 3).

Comparing both GPA subsets, patients with GPA HE were 5.5 years younger at diagnosis (49.8 vs. 55.3 years, *p* = 0.03) but did not differ in clinics, with constitutional symptoms the most common, followed by renal and respiratory involvement (Table 3). Treatments used in both subsets were also the same, apart from the borderline significant, more prevalent use of oral glucocorticosteroids for the remission induction phase in GPA NE (54.76% vs. 71.53%, *p* = 0.04). The median CRP level at diagnosis was 34% higher in GPA HE than GPA NE (83 vs. 49 mg/l, *p*  < 0.01).

### 3.5. Clinical Manifestations and ANCA Specificity Distinguish EGPA from GPA with Increased Blood Eosinophilia

**Figure 2:**
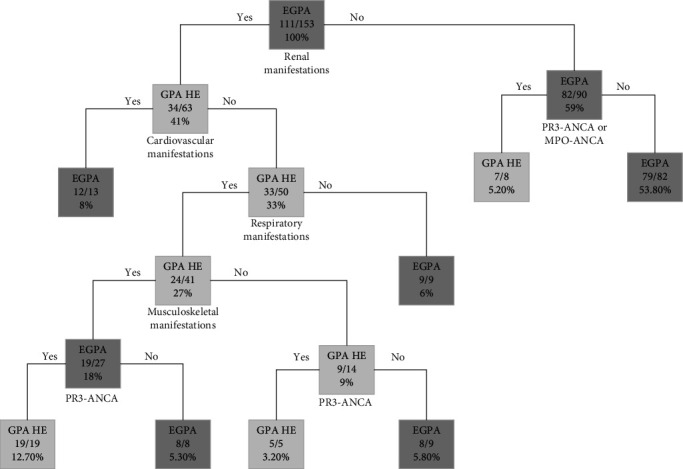
Classification tree for differentiating EGPA and GPA HE. The highest row in every node is the diagnosis; the middle row is the number of classified patients/total number of patients in the node; the lowest row is the percentage of the entire sample. Abbreviations: EGPA, eosinophilic granulomatosis with polyangiitis; GPA HE, granulomatosis with polyangiitis and elevated blood eosinophil count.

The classification tree model aiming to differentiate EGPA and GPA HE is depicted in Figure 2. All patients from respective subgroups were included in the analysis.

**Figure 3:**
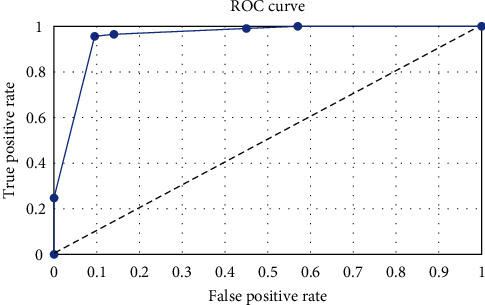
The receiver operating characteristic curve for the model differentiating patients with EGPA and GPA HE.

The final model was developed to maximize its prediction performance, resulting in a misclassification rate of 4%. Out of 42 GPA cases with increased blood eosinophilia, only two (4.76%) were assigned incorrectly, and out of 111 EGPA patients, only four (3.60%) were set incorrectly. ROC analysis was utilized to evaluate the model's performance, as shown in Figure 3. The area under the curve (AUC), sensitivity, and specificity were determined to be 92%, 96%, and 95%, respectively. The model, which consists of 15 nodes, was based on ANCA status and four types of basic clinical manifestations (renal, cardiovascular, respiratory, and musculoskeletal) as predictors.

### 3.6. Blood Eosinophilia in MPA Patients

Out of 169 MPA patients in the POLVAS registry, only 10 (5.92%) had maximal blood eosinophilia ≥400/*μ*l, with a median value of 570/*μ*l. Only two (20%) cases filled the hypereosinophilia criterion, with counts of 1,284 and 1,280/*μ*l. Six (60%) patients were female. Only one patient (10%) was PR3-ANCA positive; the remaining cases (*n* = 9, 90%) were MPO-ANCA positive. All patients had renal involvement (*n* = 11, 100%); constitutional (*n* = 8, 80%) and respiratory (*n* = 7, 70%) manifestations were also prevalent. Musculoskeletal (*n* = 3, 30%) and cutaneous (*n* = 2, 20%) symptoms were reported rarely.

## 4. Discussion

Given the rarity of EGPA, studies on large groups are scarce. The present study enrolled 111 EGPA white patients of the Polish population, accounting for 12.42% of AAV cases in the POLVAS registry.

As expected, other reports on large EGPA cohorts support our observations regarding most demographic and clinical characteristics [4, 7, 19–24], except for the gender ratio. In our EGPA group, females were more prevalent, which stays in line with reports published by Tsurkisawa et al. [19], Doubelt et al. [21], and Sokołowska et al. [9]. On the other hand, Comarmond et al. [4], Samson et al. [20], and Solans-Laqué et al. [23] reported higher or equal morbidity in males. This discrepancy might be related, e.g., to environmental or genetic factors.

The percentage of ANCA-positive cases in our dataset is also consistent with previous reports [2–4]. However, the approach to testing MPO-ANCA in negative indirect immunofluorescence (IIF) patients was not uniform among the POLVAS centers. In some cases, MPO-ANCA was tested regardless of the IIF result, while in others, it was tested only if IIF was positive. According to Tervaert et al. [25, 26], an IIF-negative result cannot rule out MPO-ANCA-positive status; therefore, the actual percentage of MPO-ANCA-positive patients may be higher.

Another issue influencing MPO-ANCA-positive EGPA percentage in this study is the unavailability of MPO-ANCA testing in some of the POLVAS centers in the past. No cases of confirmed MPO-ANCA-positive EGPA were reported between 2002 and 2007. On the other hand, since 2012, the incidence of ANCA-negative and MPO-ANCA-positive EGPA is nearly equal (data not shown).

In our study, EGPA patients did not differ regarding ANCA status in main clinical manifestations. On the other hand, comparison limited to the MPO-ANCA-positive vs. ANCA-negative subsets showed some differences, which is consistent with Moiseev et al. [27]. For instance, a higher prevalence of cardiovascular symptoms characterized the latter subset. Notably, differences in the prevalence of cardiac manifestations are the most consistently observed among large EGPA cohorts regarding ANCA status [6, 27]. However, it is important to note that cardiovascular involvement in the POLVAS cohort is likely underestimated due to the absence of routine extensive cardiac evaluation, including cardiac magnetic resonance imaging (MRI). A previous study by Dennert et al. [28] has shown that MRI can detect heart changes in over 60% of EGPA cases. Additionally, in our registry, the ANCA-negative subset was characterized by a tendency toward a higher prevalence of gastrointestinal symptoms, while in MPO-ANCA-positive patients, renal symptoms tended to be more prevalent. Although neither of these differences reached statistical significance, they were reported between ANCA-negative and ANCA-positive patients in some previous reports. For example, Healy et al. [8] demonstrated a higher prevalence of gastrointestinal symptoms in ANCA-negative EGPA, whereas renal involvement was more prevalent in ANCA-positive EGPA in studies by Comarmond et al. [4] and Sinico et al. [29].

Interestingly, in our data, ANCA-positive patients were older. It might be explained by the later age of vasculitic component onset [30, 31], since in AAVs, rises of ANCA may gain on relapse of clinical symptoms at least 6–12 months [32]. However, studies concerning the role of ANCA monitoring in AAVs were restricted to GPA and MPA patients, making it unclear whether the results can be extrapolated to EGPA [27]. Furthermore, monitoring ANCA in EGPA is useful only in those with MPO-ANCA-positive status at onset [27]. In addition, ANCA status alone is insufficient to determine the vasculitic phenotype of EGPA [27].

The next issue that merits comment on our data is the impact of the classification criteria of EGPA applied at the time of diagnosis. 1990 ACR classification criteria claim high specificity but much lower sensitivity [11], compared to the 2012 Revised International Chapel Hill Consensus nomenclature [1]. Indeed, when we stratified our patients according to applied criteria, those diagnosed after 2012 were characterized by less prevalent cardiovascular and ENT involvement and lower eosinophilia. Thus, the novel criteria likely allowed the diagnosis of milder disease, improving patients' care and future prognosis. A shift in treatment approach toward minimizing GCs use in therapy was also visible in the study, together with an increase in the use of immunosuppressive treatments in the post-2012 subgroup, according to the new recommendations [33, 34].

Another interesting issue of this study is a comparison of EGPA and GPA patients divided according to blood eosinophilia. Due to the sharing of both eosinophilic and vasculitic components, an overlap of clinical pictures has already been considered in studies on GPA subsets [12]. For the purpose of the analysis, the threshold for an elevated blood eosinophil count was established at 400/*µ*l, aligning with recent findings reported by Hartl et al. [35].

GPA subsets did not differ in clinics, but GPA NE had a lower prevalence of cutaneous and gastrointestinal manifestations than EGPA. While gastrointestinal symptoms are linked with the eosinophilic component of EGPA, cutaneous manifestations might be associated with vasculitis [6]. The difference in prevalence of gastrointestinal and cutaneous manifestations was previously shown by Iudici et al. [12]. Predominantly eosinophilic manifestations, such as respiratory or cardiovascular involvement [15, 36], were observed most commonly in EGPA patients. This study neither show a similarity between EGPA and GPA HE nor did it reveal differences between GPA HE and GPA NE in terms of neurological manifestations. In the report by Iudici et al. [12], symptoms from the central and peripheral nervous system were analyzed collectively as neurological manifestations, and no differences were observed between groups of different eosinophil counts as well. However, in the same study, peripheral and motor neuropathy analyzed separately were more prevalent in GPA with elevated blood eosinophils count than in the subset with normal eosinophil blood count.

Regarding treatments, the uniformity of GPA subsets suggests that increased eosinophilia was not influencing therapeutic approaches. Less intensive use of GCs and more common use of cyclophosphamide in the entire GPA group is not surprising and consistent with current guidelines [37].

Finally, it is worth noting that all subgroups profoundly differed in CRP levels at diagnosis, with the highest levels observed in GPA HE and the lowest in the EGPA. Interestingly, a similar pattern can be observed in the case of renal manifestations, even though the difference between subsets of GPA is on the border of significance (*p* = 0.05). As CRP is not only a nonspecific biomarker of inflammation but also contributes to forming complement C4 deposits, which play a pivotal role in developing renal manifestations [38], it can partially explain these findings.

An observation regarding the prevalence of ANCA statuses in this study is consistent with previous reports [6, 39, 40].

Since hypereosinophilia is a characteristic but not a pathognomonic feature of EGPA [41], distinguishing GPA or MPA with elevated eosinophilia from EGPA can be problematic. The main clinical features included in the 1990 ACR criteria for EGPA are hypereosinophilia, peripheral nervous system involvement, and ENT and respiratory symptoms [10]. On the other hand, the criteria introduced in 2012 are fulfilled if a patient with hypereosinophilia and asthma also has features of vasculitis or ANCA-positive status with any systemic organ involvement [1]. Therefore, both might be ineffective if applied to atypical cases of EGPA and GPA or MPA. In this study, nearly 10% of EGPA patients were PR3-ANCA positive, and 27% had renal manifestations. Furthermore, ENT and pulmonary manifestations characterized a substantial percentage of GPA patients. Therefore, the proposed algorithm effectively differentiating EGPA and GPA HE cases is a novel and interesting approach to that issue. However, its validation requires further investigation.

### 4.1. Study Limitations

Due to the retrospective, multicenter design, this study has certain limitations. First, methods used in obtaining laboratory data may vary from center to center. Second, data were collected from patients treated for many years; thus, establishing a precise definition of relapse, including vasculitic and respiratory ones, was unfeasible.

The broad categories used to classify patients in the POLVAS database make further in-depth analyses impossible. Some clinical categories encompass symptoms of significantly different pathomechanisms; for example, cutaneous manifestations include both purpura and skin nodules.

The POLVAS register did not collect data regarding exposure to environmental factors, infections, or drugs that might be a culprit of the disease. Finally, all patients included were white Caucasians, which is associated with the structure of the Polish population.

## 5. Conclusions

ANCA-positive and ANCA-negative EGPA patients did not differ in clinics, supporting their classification as a joint group. Nevertheless, cardiovascular symptoms were reported more often in ANCA-negative than MPO-ANCA-positive patients. Therefore, it seems that not the ANCA presence in general but MPO-ANCA status is critical for differentiating EGPA in terms of clinical and likely therapeutic strategy.

Moreover, since EGPA and GPA with elevated blood eosinophilia share some clinical traits, diagnostic confusion may lead to inappropriate therapy, e.g., some “eosinophilic” GPA patients may benefit from anti-IL-5 strategy, usually not recommended in AVV other than EGPA.

The proposed model distinguishing EGPA from GPA with elevated eosinophilia seems effective and may be helpful in clinics but must be validated in large AAV cohorts.

Finally, we have demonstrated that 2012 Revised Chapel Hill nomenclature may aid in identifying patients with less severe EGPA forms and, thus, likely benefit patients' outcomes.

## Figures and Tables

**Table 1:** Demographic and clinical characteristics of EGPA patients divided according to ANCA status.

Variable	ANCA+ (*n* = 45)	ANCA– (*n* = 66)	*p*-Value
Demographic characteristics			

Age at diagnosis, years	51.1 (40.2–59.0)	41.08 (33.1–51.8)	<0.01 ^*∗*^
Time of observation, years	5.1 (4.0–6.2)	7.6 (3.3–4.6)	0.03^*∗*^
Female gender, number (%)	29 (64.44)	48 (72.72)	0.38
Smoking in the past, number (%)	4 (8.89)	8 (12.12)	0.38

Clinical manifestations			

Constitutional symptoms	40 (88.89)	59 (89.39)	0.80
Musculoskeletal	26 (57.78)	29 (43.94)	0.17
Cutaneous	23 (51.11)	36 (54.55)	0.73
Ocular	4 (8.89)	3 (4.55)	0.59
ENT	38 (84.44)	54 (81.82)	0.85
Respiratory	43 (95.56)	64 (96.97)	0.36
Cardiovascular	16 (35.56)	31 (46.97)	0.21
Gastrointestinal	11 (24.44)	21 (31.82)	0.38
Renal	15 (33.33)	15 (22.73)	0.21
Central nervous system	4 (8.89)	7 (10.61)	0.998
Neurological	24 (53.33)	29 (43.94)	0.37
Flare-ups, number	1 (0–2)	2 (1–3)	<0.01 ^*∗*^
Flare-ups, number/time of observation, years	0.18 (0.00–0.33)	0.26 (0.14–0.35)	0.20
Patients with flare-ups requiring hospital admission, number	10 (22.22)	17 (25.76)	0.56

Remission induction therapy			

Oral glucocorticosteroids	42 (93.33)	61 (92.42)	0.07
Glucocorticosteroid pulses	27 (60.00)	47 (71.21)	0.13
Cyclophosphamide	18 (40.00)	31 (46.97)	0.37
Azathioprine	3 (6.67)	11 (16.67)	0.18
Methotrexate	11 (24.44)	10 (15.15)	0.26
Mycophenolate mofetil	1 (2.22)	4 (6.06)	0.60
The cumulative time of glucocorticosteroid treatment, years	3.50 (2.00–7.50)	5.00 (3.00–7.50)	0.20
The cumulative dose of intravenous glucocorticosteroids (g)	1.45 (0.00–6.70)	2.60 (0.00–8.00)	0.78

Maintenance therapy			

Oral glucocorticosteroids	32 (71.11)	37 (56.06)	0.25
Azathioprine	16 (35.56)	18 (27.27)	0.80
Methotrexate	14 (31.11)	14 (21.21)	0.87
Mycophenolate mofetil	5 (11.11)	11 (16.67)	0.12

Laboratory parameters			

Blood eosinophilia (/*μ*l)^&^	4,100 (2,000–7,676)	5,608 (3,000–9,454)	0.21
Max. CRP at diagnosis (mg/l)	33.0 (11.0–60.0)	21.0 (10.0–50.0)	0.54

Glucocorticosteroid doses were adjusted to methylprednisolone. Categorical variables are presented as numbers (percentages), continuous variables as median, and 0.25–0.75 interquartile range. ^&^Data available in 38 (80, 85%) ANCA-positive and 54 (78, 26%) ANCA-negative patients. Statistically significant differences are highlighted with an asterisk. Abbreviations: ANCA, anti-neutrophil cytoplasmic antibodies; CRP, C-reactive protein; EGPA, eosinophilic granulomatosis with polyangiitis; and ENT, ear/nose/throat.

**Table 2:** Demographic and clinical characteristics of EGPA patients diagnosed before and after the 2012 year.

Variable	≤2012 (*n* = 70)	>2012 (*n* = 41)	*p*-Value
Demographic characteristics			

Age at diagnosis, years	41.3 (33.1–52.4)	49.17 (40.3–58.4)	<0.01 ^*∗*^
Female gender, number (%)	51 (72.86)	26 (63.41)	0.30
Smoking in the past, number (%)	4 (5.71)	8 (19.51)	0.97

Clinical manifestations			

Constitutional symptoms	65 (92.86)	34 (82.93)	0.23
Musculoskeletal	31 (44.29)	24 (58.54)	0.11
Cutaneous	37 (52.86)	21 (51.22)	0.96
Ocular	3 (4.29)	4 (9.76)	0.25
ENT	64 (91.43)	28 (68.29)	<0.01 ^*∗*^
Respiratory	69 (98.57)	38 (92.68)	0.62
Cardiovascular	35 (50.00)	12 (29.27)	0.04^*∗*^
Gastrointestinal	20 (28.57)	12 (29.27)	0.89
Renal	20 (28.57)	10 (24.39)	0.64
Central nervous system	6 (8.57)	5 (12.20)	0.52
Neurological	36 (51.43)	17 (41.46)	0.36
Flare-ups, number	3 (1–3)	0 (0–1)	<0.01 ^*∗*^
Flare-ups, number/time of observation, years	0.28 (0.18–0.35)	0.00 (0.00–0.33)	0.03^*∗*^
Patients with flare-ups requiring hospital admission, number	15 (21.43)	12 (29.27)	0.26

Remission induction therapy			

Oral glucocorticosteroids	69 (98.57)	34 (82.93)^*α*^	<0.01 ^*∗*^
Glucocorticosteroid pulses	56 (80.00)	18 (43.90)	<0.01 ^*∗*^
Cyclophosphamide	31 (44.29)	18 (43.90)	0.75
Azathioprine	11 (15.71)	3 (7.32)	0.25
Methotrexate	8 (11.43)	13 (31.71)	<0.01 ^*∗*^
Mycophenolate mofetil	0 (0.00)	5 (12.20)	<0.01 ^*∗*^
The cumulative time of glucocorticosteroid treatment, years	7.00 (5.00–10.00)	2.00 (1.00–4.00)	<0.01 ^*∗*^
The cumulative dose of intravenous glucocorticosteroids (g)	4.20 (0.00–8.60)	1.45 (0.00–6.00)	0.37

Maintenance therapy			

Oral glucocorticosteroids	37 (52.86)	32 (78.05)	0.08
Azathioprine	19 (27.14)	15 (36.59)	0.36
Methotrexate	9 (12.86)	19 (46.34)	<0.01 ^*∗*^
Mycophenolate mofetil	5 (7.14)	11 (26.83)	0.09

Laboratory parameters			

Blood eosinophilia (/*μ*l)^*β*^	4,946 (2,400–8,253)	3,200 (358–5,800)	<0.01 ^*∗*^
Max. CRP at diagnosis (mg/l)	23.9 (10.0–58.0)	22.7 (10.0–60.0)	0.91
ANCA-positive, *n* (%)	23 (32.86)	22 (53.66)	0.11

Categorical variables are presented as numbers (percentages), continuous variables as median, and 0.25–0.75 interquartile range. ^*α*^Among seven cases that did not receive oral GCs in the remission induction phase, all but one were administered intravenous GCs and the remaining one cyclophosphamide; in the maintenance therapy, all received oral GCs. ^*β*^Data available in 55 (78.57.%) and 26 (63.41%) EGPA patients in the first and second subgroups, respectively. Statistically significant differences are highlighted with an asterisk. Abbreviations: ANCA, anti-neutrophil cytoplasmic antibodies; CRP, C-reactive protein; EGPA, eosinophilic granulomatosis with polyangiitis; and ENT, ear/nose/throat.

**Table 3:** Demographic and clinical characteristics of EGPA and GPA patients divided according to eosinophilia.

Variable	EGPA, *n* = 111	GPA HE, *n* = 42	GPA NE, *n* = 281	*p*-Value EGPA vs. GPA HE	*p*-Value EGPA vs. GPA NE	*p*-Value GPA HE vs. GPA NE
Demographic characteristics						

Age at diagnosis, years	45.0 (34.8–54.9)	49.8 (35.0–56.0)	55.3 (41.5–51.8)	0.44	<0.01 ^*∗*^	0.03^*∗*^
Time of observation, years	6.61 (4.12–9.70)	7.00 (4.91–13.76)	5.10 (2.25–9.59)	0.30	0.06	0.04^*∗*^
Female gender, number	77 (69.37)	16 (38.10)	151 (53.74)	<0.01 ^*∗*^	0.03^*∗*^	0.08
Smoking in the past	47 (42.34)	11 (26.19)	62 (22.06)	0.60	0.97	0.67

Clinical manifestations						

Constitutional symptoms	99 (89.19)	39 (92.86)	234 (83.27)	0.38	0.59	0.17
Musculoskeletal	55 (49.55)	30 (71.43)	157 (55.87)	0.02^*∗*^	0.23	0.08
Cutaneous	58 (52.25)	16 (38.10)	85 (30.25)	0.22	<0.01 ^*∗*^	0.39
Ocular	7 (6.32)	10 (23.81)	76 (27.05)	<0.01 ^*∗*^	<0.01 ^*∗*^	0.78
ENT	92 (82.88)	27 (64.29)	221 (78.65)	0.07	0.86	0.61
Respiratory	107 (96.39)	30 (71.43)	215 (76.51)	<0.01 ^*∗*^	<0.01 ^*∗*^	0.60
Cardiovascular	47 (42.34)	3 (7.14)	32 (11.39)	<0.01 ^*∗*^	<0.01 ^*∗*^	0.57
Gastrointestinal	32 (28.82)	6 (14.29)	29 (10.32)	0.12	<0.01 ^*∗*^	0.61
Renal	30 (27.03)	34 (80.95)	181 (64.41)	<0.01 ^*∗*^	<0.01 ^*∗*^	0.05
Central nervous system	11 (9.91)	4 (9.52)	34 (12.10)	0.77	0.58	0.81
Neurological	53 (47.75)	9 (21.43)	60 (21.35)	0.01^*∗*^	<0.01 ^*∗*^	0.89
Flare-ups	2 (0–3)	1 (0–2)	1 (0–2)	0^*∗*^	<0.01 ^*∗*^	0.95
Flare-ups, number/time of observation, years	0.26 (0.00–0.35)	0.08 (0.00–0.25)	0.10 (0.00–0.31)	<0.01 ^*∗*^	0.01^*∗*^	0.53
Patients with flare-ups requiring hospital admission	27 (24.32)	22 (52.38)	148 (52.67)	<0.01 ^*∗*^	<0.01 ^*∗*^	0.93

Remission induction						

Oral glucocorticosteroids	103 (92.79)	23 (54.76)	201 (71.53)	<0.01 ^*∗*^	<0.01 ^*∗*^	0.04^*∗*^
Glucocorticosteroid pulses	74 (66.67)	31 (73.81)	221 (78.65)	0.51	0.01^*∗*^	0.61
Cyclophosphamide	49 (44.14)	32 (76.19)	240 (85.41)	<0.01 ^*∗*^	<0.01 ^*∗*^	0.19
Azathioprine	14 (12.61)	1 (2.38)	13 (4.63)	0.11	<0.01 ^*∗*^	0.79
Methotrexate	21 (18.92)	1 (2.38)	26 (9.25)	0.02^*∗*^	0.01^*∗*^	0.23
Mycophenolate mofetil	5 (4.50)	0 (0.00)	7 (2.49)	0.38	0.47	0.64
Rituximab	0 (0.00)	11 (26.19)	50 (17.79)	<0.01 ^*∗*^	<0.01 ^*∗*^	0.28
Blood apharesis	0 (0.00)	5 (11.90)	31 (11.03)	0.01^*∗*^	<0.01 ^*∗*^	0.92
The cumulative time of glucocorticosteroid treatment, years	4.00 (2.00–7.50)	3.00 (2.00–8.00)	4.00 (2.00–8.00)	0.66	<0.01 ^*∗*^	0.75
The cumulative dose of intravenous glucocorticosteroids (g)	1.90 (0.00–8.00)	6.50 (2.00–16.50)	7.1 (3.60–14.40)	<0.01 ^*∗*^	<0.01 ^*∗*^	0.48

Maintenance therapy						

Oral glucocorticosteroids	69 (62.16)	35 (83.33)	235 (83.63)	0.88	0.53	0.08
Azathioprine	34 (30.63)	15 (35.71)	123 (43.77)	0.66	0.99	0.51
Methotrexate	28 (25.23)	8 (19.05)	66 (23.49)	0.12	0.046	0.74
Mycophenolate mofetil	16 (14.41)	6 (14.29)	65 (23.13)	0.63	0.69	0.32
Cyclophosphamide	2 (1.80)	5 (11.90)	25 (8.90)	0.02^*∗*^	0.02^*∗*^	0.68

Laboratory parameters						

ANCA-positive	45 (40.54)	41 (97.62)	246 (87.54)	<0.01 ^*∗*^	<0.01 ^*∗*^	0.06^*∗*^
MPO-ANCA positive	26 (23.42)	2 (4.76)	17 (6.05)	0.01^*∗*^	<0.01 ^*∗*^	0.99
PR3-ANCA positive	11 (9.91)	39 (92.86)	214 (76.16)	<0.01 ^*∗*^	<0.01 ^*∗*^	0.08
Blood eosinophilia (number/*μ*l)	4,992 (2,500–8,253)	700 (465–1,294)	100 (50–170)	<0.01 ^*∗*^	<0.01 ^*∗*^	—
Max. CRP at diagnosis (mg/l)	23.9 (10.0–58.0)	83.0 (45.0–172.0)	49.0 (14.0–114.0)	<0.01 ^*∗*^	<0.01 ^*∗*^	<0.01 ^*∗*^

Categorical variables are presented as numbers (percentages), continuous variables as median, and 0.25–0.75 interquartile range. Statistically significant differences are highlighted with an asterisk. Abbreviations: ANCA, anti-neutrophil cytoplasmic antibodies; MPO-ANCA, anti-myeloperoxidase antibodies; PR3-ANCA, anti-proteinase three antibodies; CRP, C-reactive protein; EGPA, eosinophilic granulomatosis with polyangiitis; ENT, ear/nose/throat; GPA HE, granulomatosis with polyangiitis and increased eosinophil blood count; and GPA NE, granulomatosis with polyangiitis and a normal eosinophil blood count.

## Data Availability

The data underlying this article will be shared upon reasonable request from the corresponding author.

## Supplementary Materials

Supplementary Materials The comprehensive list of symptoms reported in the POLVAS database was included in Supplementary Data S1.
