# Supplementary material for: Clinical Characteristics of EGPA Patients in Comparison to GPA Subgroup with Increased Blood Eosinophilia from POLVAS Registry
Source: J Immunol Res. 2024 Apr 25;2024:4283928. doi: 10.1155/2024/4283928 (PMC11065486; doi:10.1155/2024/4283928)
Supplement: Supplementary Materials — The comprehensive list of symptoms reported in the POLVAS database was included in Supplementary Data S1. [file 4283928.f1.docx]

**Supplementary Data S1**

**List of symptoms and signs in respective manifestations categories in the POLVAS database.**

**Constitutional symptoms** comprised syncope, fatigue, fever over 38°C, weight loss over 2 kg, night sweats, cervical lymphadenopathy over 1,5 cm, and lymphadenopathy in minimum of two anatomical areas.

**Musculoskeletal manifestations** included myalgia, weakness or pain of lower extremities, morning stiffness, and arthritis.

**Cutaneous manifestations** were defined as papular or papulo-macular rash, acne or vesicular changes, reticular livedo, erythema nodosum, palmar and plantar rash, epidermal desquamation on fingertips, palmar and plantar edema, erythema multiforme, palpable purpura, skin ulcers, widespread skin necrosis, splinter sign, skin nodules, urticaria, and pathergy.

**Ocular manifestations** included lacrimation or lacrimal gland enlargement, blepharitis, conjunctivitis, optic neuritis, choroiditis, corneitis, sudden sight loss, retinal vasculitis, retinal effusions, retinal hemorrhages, venous or arterial thrombosis, orbital pseudotumor, orbital wall defects, blindness.

**Ear/nose/throat (ENT)** involvement was defined as jaw or tongue claudication, paranasal sinusitis, nosebleeds, nasal discharge, ear or nose chondritis, mastoiditis, otitis media, hearing loss, saddle nose, disorders of the nasal mucosa (ulceration, crusts etc.), damage or perforation of nasal septum, oral cavity ulcerations, reddened and chapped lips, strawberry tongue/mouth and throat mucosal congestion, salivary glands enlargement, subglottic stenosis.

**Respiratory manifestations** comprised dyspnea, dry cough, wet cough with purulent sputum, hemoptysis, lung fibrosis, wheezing, nodules or cavities in lungs, lung infiltration, diffuse alveolar hemorrhage, respiratory disorders requiring oxygen therapy, respiratory failure requiring intubation, pleural effusion, or pain.

**Cardiovascular manifestations** comprised limb claudication, carotidynia, Raynaud phenomenon, stable angina, confirmed myocardial infarction, cardiomyopathy, myocarditis, pericarditis, clot in heart chambers, valvular defect which occurred in adulthood, congestive heart failure, arrhythmias, an abnormal test of a temporal artery or any other artery, pulse deficit in brachial artery, systolic pressure difference between upper extremities >10mg Hg, murmur over a subclavian artery or abdominal aorta, and venous thrombosis.

**Digestive system manifestations** were defined as abdominal pain, diarrhea, gastrointestinal bleeding, jaundice, acute or chronic pancreatitis, chronic peritonitis, colitis confirmed by endoscopic examination, postprandial/ischemic abdominal pain, mesenteric ischemia, and bowel infarction/resection.

**Renal manifestations** comprised renal insufficiency requiring hemodialysis or transplantation, dysuria, urinary tract obstruction, proteinuria, and pain of the lumbar area.

**Central nervous system manifestations** comprised transient ischemic attack or cerebrovascular event, photophobia, confusion of organic etiology, convulsions, meningitis, head skin tenderness, and headache in frontal, temporal, occipital area or back of the neck.

In contrast, other neurologic manifestations included spinal cord damage, cranial nerves involvement, neuropathy (motor, sensory, mono- or polyneuropathy), and mononeuritis multiplex.
